# The Homœopathic Envoy

**Published:** 1894-10

**Authors:** 


THE HOMŒOPATHIC ENVOY. E. P. Anshutz, P. O. Box 921, Philadelphia, Pa. Monthly. Price, 25 cents a year.

This periodical is a clever defense and argument for Homœopathy. It is published once a month, of quarto size, and eight pages. It is intended especially for the laity. Its editorials are admirable and worthy the careful attention of practitioners of homœopathic medicine as well as the laity. It is useful to those physicians who wish to put into the hands of their patients something to read upon the great principles of the new school of medicine.

After repeated perusal of its pages, the editor of this journal is able to warmly recommend it.

